# Nymphalid eyespots are co-opted to novel wing locations following a similar pattern in independent lineages

**DOI:** 10.1186/s12862-015-0300-x

**Published:** 2015-02-14

**Authors:** Sandra R Schachat, Jeffrey C Oliver, Antónia Monteiro

**Affiliations:** Department of Biochemistry, Molecular Biology, Entomology and Plant Pathology, Mississippi State University, Mississippi State, MS 39762 USA; Department of Paleobiology, Smithsonian Institution, Washington, DC 20013 USA; Department of Integrative Biology, Oregon State University, Corvallis, OR 97331 USA; Department of Ecology & Evolutionary Biology, Yale University, New Haven, CT 06520 USA; Department of Biological Sciences, National University of Singapore, 117543 Singapore, Singapore; Yale-NUS College, 138614 Singapore, Singapore

**Keywords:** Serial homology, Correlation analysis, Ancestral states, Phylogeny, Wing patterns

## Abstract

**Background:**

Variation in the number of repeated traits, or serial homologs, has contributed greatly to animal body plan diversity. Eyespot color patterns of nymphalid butterflies, like arthropod and vertebrate limbs, are an example of serial homologs. These eyespot color patterns originated in a small number of wing sectors on the ventral hindwing surface and later appeared in novel wing sectors, novel wings, and novel wing surfaces. However, the details of how eyespots were co-opted to these novel wing locations are currently unknown.

**Results:**

We used a large data matrix of eyespot/presence absence data, previously assembled from photographs of contemporary species, to perform a phylogenetic investigation of eyespot origins in nine independent nymphalid lineages. To determine how the eyespot gene regulatory network acquired novel positional information, we used phylogenetic correlation analyses to test for non-independence in the origination of eyespots. We found consistent patterns of eyespot gene network redeployment in the nine lineages, where eyespots first redeployed from the ventral hindwing to the ventral forewing, then to new sectors within the ventral wing surface, and finally to the dorsal wing surface. Eyespots that appeared in novel wing sectors modified the positional information of their serial homolog ancestors in one of two ways: by changing the wing or surface identity while retaining sector identity, or by changing the sector identity while retaining wing and surface identity.

**Conclusions:**

Eyespot redeployment to novel sectors, wings, and surfaces happened multiple times in different nymphalid subfamilies following a similar pattern. This indicates that parallel mutations altering expression of the eyespot gene regulatory network led to its co-option to novel wing locations over time.

**Electronic supplementary material:**

The online version of this article (doi:10.1186/s12862-015-0300-x) contains supplementary material, which is available to authorized users.

## Background

Animal bodies are often made up of repeated units, or serial homologs, which vary in number, size, shape, and position and largely contribute to body plan diversity. Examples of serial homologs include vertebrate limbs [1] and teeth [2]; insect limbs [3], bristles [4,5], and trichomes [6,7]; and nymphalid butterfly eyespots [8]. In many animal lineages, the development of serial homologs and other complex traits is mediated via gene regulatory networks [9]. Serial homologs develop using a largely shared network [10]. However, in many of the examples above, it is still unclear how the gene regulatory network, responsible for the development of each serial homolog, became deployed at each body location and gained individuation from the other units to generate body plan diversity.

**Figure 1:**
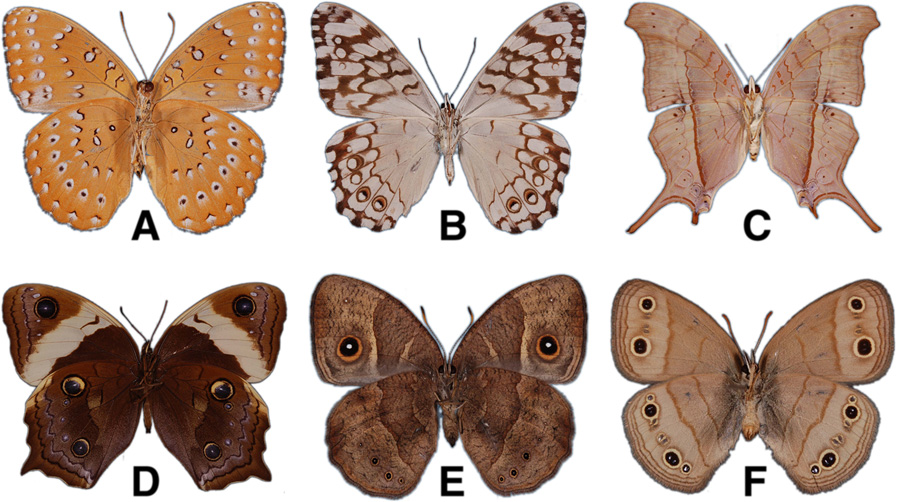
**Examples of eyespot diversity in nymphalid butterflies. (A)** *Hamanumida daedalus* (YPM ENT 422238). **(B)** *Hamadryas februa* (YPM ENT 815633). **(C)** *Marpesia eleuchea* (YPM ENT 815891). **(D)** *Neorina crishna* (YPM ENT 407679). **(E)** *Bicyclus anynana* (YPM ENT 814497). **(F)** *Megisto cymela* (YPM ENT 405705). All photographs show the ventral wing surface of female butterflies; all specimens represent taxa investigated in this study and are housed in the Entomology Collection of the Yale Peabody Museum of Natural History.

Here we focus on unraveling the evolution of nymphalid butterfly eyespot number. Variation in eyespot number and position contributes to the morphological diversity of this clade (Figure 1). Earlier hypotheses, based on the nymphalid groundplan model [11], suggested that eyespot number diversity could have arisen from a primitive state where eyespots were present across all wing sectors [8,12]. Recent advances, however, show that Nymphalid eyespots originated during the late Cretaceous in a few sectors on the ventral surface of the hindwing [13,14]. This means that the eyespot gene regulatory network originated with positional information coding for a particular wing (hindwing), a particular surface (ventral surface), and particular sector identities (Rs, M1, M2, M3, and Cu1). Subsequently, perhaps due to evolution in the regulation of key network genes [5,15], the network was redeployed in novel wings, surfaces, and wing sectors [13,14], but the details of these redeployments have not yet been described.

Here we take a phylogenetic approach to examine how eyespots evolved at novel wing locations. We used correlation analyses to elucidate patterns of independence and non-independence across pairs of eyespots, and therefore, the patterns of eyespot deployment on the wing. We used ancestral state reconstructions to evaluate an iterative series of eyespot evolution models, allowing tests of potential pathways through which eyespots may be deployed across different wing locations. These models provide a test for whether the origin of an eyespot in one wing location is contingent on the presence of another eyespot in a different wing location. Comparison among models of evolutionary change thus allow us to evaluate support for the possibility that regulatory information coding for the more recent (i.e. “derived”) eyespot evolved from regulatory information that coded for the older (i.e. “ancestral”) eyespot. In contrast, those cases in which eyespot origins are not contingent on the presence of another eyespot on the wings may indicate that independent enhancers, or regulatory sequences, have evolved for each eyespot. By examining the evolutionary history of multiple eyespots, our goal is to understand the relationship between pre-existing positional information for extant eyespots and novel positional information required to deploy eyespots to new locations. Furthermore, by evaluating these models in multiple lineages of nymphalid butterflies we can assess the degree to which there are shared pathways by which novel positional information evolves to generate diversity in butterfly eyespot number and location.

## Methods

### Data collection and ancestral state reconstructions

We collected character data from 394 nymphalid species based on phylogenetic sampling of previous studies [16,17]. The majority of specimens are from holdings in the Yale Peabody Natural History Museum, the American Museum of Natural History, and the Harvard Museum of Comparative Zoology. For each species, we scored eyespot presence/absence for all wing surfaces (the dorsal and ventral surfaces of the forewing and hindwing), for a grand total of 38 different wing sectors for a single side of the body (Additional file 1). We scored eyespots for each of the sexes, and used two specimens per sex when possible. An eyespot is defined as a circular pattern element containing two or more concentric rings of color [8]. Additional pattern elements, such as patches and single-color spots, were not used in these analyses. All patterns were scored from photographs available at www.lepdata.org. We used the estimate of nymphalid phylogeny published by Wahlberg et al. [17].

### Identification of independent clades for analysis

**Figure 2:**
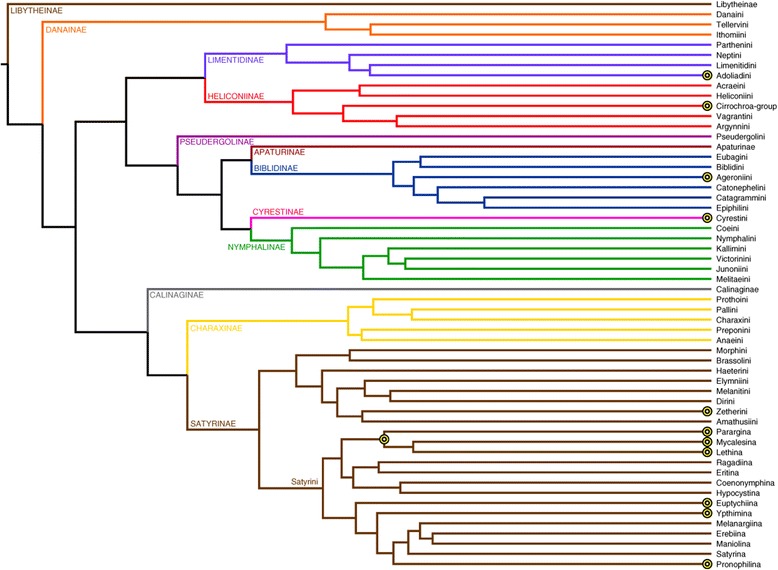
**A phylogeny of the Nymphalidae showing all tribes (and subtribes within the tribe Satyrini), modified from** **[** 17 **].** The clades analyzed in this study are marked by an eyespot; because Parargina, Mycalesina, and Lethina form a clade, they were analyzed together, as indicated by the eyespot at the node shared by these three subtribes. Colored branches represent different subfamilies.

We analyzed eyespot number evolution separately across nine clades, belonging to five different subfamilies of Nymphalidae (Figure 2). The number of eyespots present on female butterflies was totaled for each species sampled. We identified the top 50 species with the highest number of eyespots (13 or more). Of these 50 species, 18 are interspersed through 18 different clades. The other 32 are clustered into 9 clades, each of which contains at least two species with 13 or more eyespots (Additional file 2: Table S1; Figure 2). For each clade, separate eyespot presence/absence matrices were retained for each eyespot in both males and females, for a total of 76 matrices per clade. Sexes were analyzed separately.

### Eyespot models of evolution

For each clade, we restricted our analyses to those pairs of eyespots that were inferred to have a shared history in the clade being investigated. A shared history was inferred when both eyespots had a higher likelihood of being present than absent in at least one shared ancestral node. Ancestral state likelihoods were calculated in Mesquite [18], using an asymmetrical (2-parameter) likelihood model. For each pair of eyespots identified as having a shared history, we categorized one eyespot as “ancestral” (the eyespot that evolved first) and the other eyespot as “derived”. To objectively categorize the eyespots, we again used the likelihoods of presence/absence for each eyespot using an asymmetrical likelihood model in Mesquite [18]. If one eyespot had a greater likelihood of being present at a node ancestral to the “shared node” identified above, while the other eyespot had a greater likelihood of being absent at that ancestral node, the first eyespot was categorized as the ancestral eyespot. When the deepest node where each eyespot was more likely present than absent was the same for each eyespot, the eyespot with the greater likelihood of being present was categorized as the ancestral eyespot.

**Figure 3:**
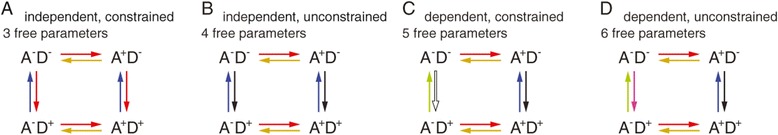
**Four models of eyespot evolution used to identify correlated eyespots. (A)** Independent constrained model, in which transition rates in an eyespot are independent of the state of the other eyespot and the origination rates for the two eyespots are set to equal; **(B)** independent unconstrained model, in which transition rates in an eyespot are independent of the state of the other eyespot; **(C)** dependent constrained model, in which the transition rates of the derived eyespot are conditional on the state of the ancestral eyespot and in which the derived eyespot may only evolve when ancestral eyespot is present (the transition rate indicated by white arrow is set to zero). **(D)** dependent unconstrained model, in which the derived eyespot may originate regardless of whether the ancestral eyespot is present or absent. Legend: A^−^: ancestral eyespot absent; A^+^: ancestral eyespot present; D^−^: derived eyespot absent; D^+^: derived eyespot present. In each panel, colors of arrows indicate unique rate parameters. Models increase in complexity, i.e., in the number of different rates of transition between character states (arrows of different colors) that have to be estimated from the data from left (model A; three rates) to right (model D; 6 rates).

After categorizing eyespots as ancestral or derived, we employed an iterative model comparison approach, based on estimated rates of eyespot gains. “Origination rates” for each eyespot are defined as the rate of transition from “absent” to “present” for an individual eyespot character. All analyses were calculated using the Discrete module of BayesTraits [19], in which we estimated the maximum likelihood ancestral states under a variety of evolutionary models (see below for model details). In all cases, simpler nested models were rejected when more complex models had likelihood scores 2 log likelihood units higher than the simpler models. Multiple instantaneous transitions were never allowed. We first compared the likelihoods of two models: (1) an independent model of evolution, where gains and losses of one eyespot were independent of the state of the other eyespot, but in which both eyespots were constrained to the same origination rate (Figure 3A; the simpler model); and (2) an independent model of evolution where the origination rates of one eyespot were not constrained to equal the origination rates of the other eyespot (Figure 3B; the more complex model). For those pairs of eyespots in which the more complex independent unconstrained model provided a better fit, we then performed another model comparison: between it (Figure 3B; now the simpler model) and a dependent constrained model of evolution, where the transition rates between present and absent for the derived eyespot were conditional on the state of the ancestral eyespot *and* the transition rate from absent to present in the derived eyespot when the ancestral eyespot was absent was set to zero (Figure 3C; the more complex model). This latter model restricts the origin of derived eyespots to those lineages in which the ancestral eyespot is present, as expected if the derived eyespot is a re-deployment of the ancestral eyespot. For those pairs of eyespots in which the more complex dependent constrained model provided a better fit, we performed one additional model comparison. We compared the dependent constrained model as described above (Figure 3C; now the simpler model) to a dependent unconstrained model, where gains of the derived eyespot were possible in the absence of the ancestral eyespot (Figure 3D; the most complex model). This last comparison effectively tests if the gain of the derived eyespot in the absence of the ancestral eyespot is likely, a condition that is not congruent with a model of re-deployment, but instead supports *de novo* evolution of positional information for the eyespot gene network. Only those pairs of eyespots for which we failed to reject the dependent constrained model (i.e. the rate of gain of the derived eyespot in the absence of the ancestral eyespot is not significantly different from zero) were identified as pairs that fit a model of network re-deployment via modification of pre-existing positional information within the network.

## Results

### Eyespot models of evolution

The eyespots of extant Nymphalidae have three kinds of positional information: wing (forewing vs. hindwing), wing surface (dorsal vs. ventral), and wing sector. Because the first eyespots arose on only one wing, on only one wing surface, and only in certain wing sectors [13], these three positional identities were present when Nymphalid eyespots first arose. In this study, eyespots are assumed to redeploy to new locations by acquiring only one new kind of positional information at a time. Therefore, the eyespot pairs used for analyses were those that differ only in wing identity (same wing sector, same wing surface), those that differ only in wing surface identity (same wing sector, same wing), and those that differ only in wing sector identity (same wing, same wing surface).

**Figure 4:**
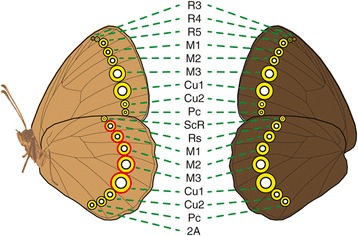
**Illustration of all the wing sectors where at least one eyespot was found in species scored for this study, and correspondent eyespot sector nomenclature.** The five eyespots identified by [13] as having appeared first are outlined in red. The ventral surface is rendered in light brown, on the left; the dorsal surface is rendered in dark brown, on the right.

**Figure 5:**
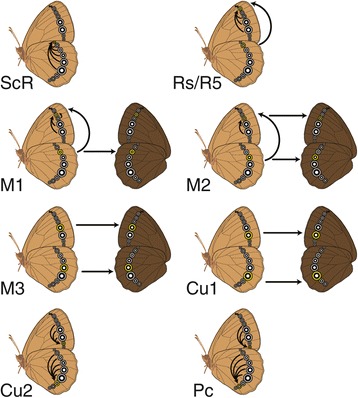
**Significant correlations between pairs of eyespots identified in this study, organized by derived eyespot.** The arrow points from the ancestral eyespot to the derived eyespot, which is colored yellow. Ventral surface is light brown; dorsal surface is dark brown.

Based on a large dataset of eyespot presence/absence for ~400 genera of nymphalid butterflies [13,16], we identified the set of wing sectors that evolved eyespots at some point in time (Figure 4). We also recovered significant evidence for non-independence between eyespots on different wings, wing surfaces, and wing sectors (Additional file 3), although origins of the first 7–8 eyespots were not contingent on the presence of any other eyespots. These eyespots included Rs, M1, M2, M3, and Cu1 on the ventral hindwing and M2, M3, and Cu1 on the ventral forewing (Figure 4) [13]. Within the ventral wing surface, we found the origin of some eyespots on the forewing (M3 and Cu1, and possibly R5, M1, and M2) to be contingent on the presence of an eyespot in the homologous sector on the hindwing (Figure 5; Additional file 4: Table S2); in contrast, origins of eyespots on the ventral hindwing were not contingent on the presence of eyespots on the ventral forewing, nor were dorsal eyespot origins on either wing (forewing/hindwing) contingent on the presence of eyespots on the other wing (hindwing/forewing). We found the origins of four dorsal eyespots (M1, M2, M3, and Cu1) to be contingent on the presence of homologous ventral eyespots (Figure 5; Additional file 5: Table S3), while ventral eyespots originated at rates independent of the presence/absence of homologous eyespots on the dorsal surface. Finally, the origins of many ventral eyespots, on both the forewing and hindwing, were dependent on the presence of at least one other eyespot located in a different sector on the same wing (Figure 5; Additional file 6: Table S4); there was no evidence that origins of dorsal eyespots were contingent on the presence of other dorsal eyespots on the same wing.

### Patterns of eyespot redeployment

**Figure 6:**
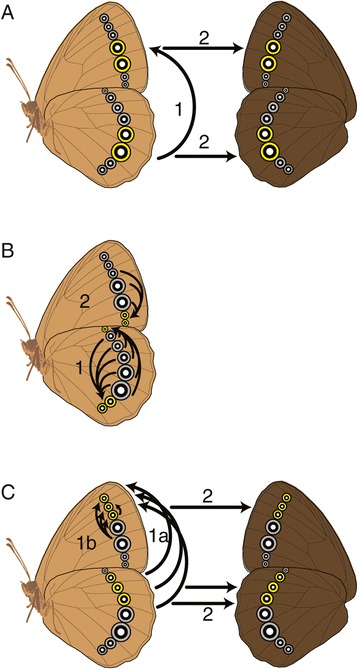
**Different patterns of eyespot redeployment: (A) between wings and surfaces (eyespots M3 and Cu1), (B) within wing surface (eyespots ScR, Cu2, and Pc), and (C) ambiguous (eyespots Rs/R5, M1, and M2).** The arrow points from the ancestral eyespots to the derived eyespots. The derived eyespots in each schematic are colored yellow. Ventral surface is light brown; dorsal surface is dark brown.

We recovered four types of redeployment for the eyespot gene network: the first involving deployment of centrally located wing eyespots between fore and hindwings (Figure 6A); the second involving deployment of the same central eyespots between the same wing sector in dorsal and ventral surfaces (Figure 6A); the third involving a different sub-set of eyespots originating via network redeployment from sectors on the same wing surface (Figure 6B); and a fourth, involving a different sub-set of eyespots originating via any of the redeployments above (Figure 6C).

Eyespots in sectors M3 and Cu1 (Figure 4) belong to the first two categories (Figure 6A). Previous work estimated that the first eyespots appeared only on the hindwing [13], and if we are correct in our assumption that eyespot redeployment occurs only by step-wise changes in positional information, then the M3 and Cu1 eyespots on the ventral forewing must have originated as a modification of the positional information coding for the M3 and Cu1 eyespots on the ventral hindwing. Positive correlation data (Additional file 5: Table S3) also supports the inference that M3 and Cu1 eyespots originated on the dorsal surface from modification of positional information coding for their ventral serial homologs.

Eyespots in sectors ScR, Cu2, and Pc belong to the third category, where eyespots are redeployed to different wing sectors within a single wing surface (Figure 6B): our results indicate that these eyespots originate from modifications of positional information coding for eyespots present in other sectors on the same wing.

The origin of eyespots R5/Rs, M1, and M2 remains ambiguous, as we recovered support for models that fit in the first, second, and third categories. At present we cannot determine whether these eyespots originate as modification of positional information coding for eyespots in other wings, wing surfaces, or from the M3 and Cu1 eyespots within the same wing surface (Figure 6C).

Within wing surfaces, eyespots do not appear sequentially in adjacent wing sectors. For example, the origin of eyespots in the Pc wing sector is never contingent on the presence of eyespots in the adjacent Cu2 wing sector.

None of the significant correlations that we recovered suggest any differences in patterns of ventral eyespot redeployment among nymphalid subfamilies, tribes, and subtribes (Additional file 4: Tables S2, Additional file 5: Table S3, Additional file 6: Table S4). The few cases of non-independence in dorsal eyespot origins are also consistent among all lineages examined in that they show that, for both wings, only eyespots in the sectors Rs, M1, M2, M3, and Cu1 redeployed from the ventral to the dorsal wing surface.

## Discussion

Here we document how nymphalid butterfly eyespot number evolved over the past ~90 million years. A recent study showed that eyespots originated in a few wing sectors on the hindwing [13]. Here we extend that study by showing that these eyespots gradually redeployed to new wings (forewing), new surfaces (the dorsal surface), and new wing sectors to produce the extant Nymphalidae eyespot diversity. In addition, these redeployments appear to have occurred in a parallel fashion across independent lineages.

We found that eyespots belonging to different wing sectors (ScR, Cu2, or Pc as opposed to M3 or Cu1) follow different patterns of gene network redeployment. ScR, Cu2, and Pc likely arise via modification of regulatory positional information that was already available for eyespots on the same wing surface, whereas M3 and Cu1 appear to arise via modification of regulatory information available for homologous eyespots on other wing surfaces. Therefore, although eyespots can appear in a continuous row within a single wing surface, they evolved positional information for several of these sectors in different ways.

### Similarities in redeployment across the phylogeny

**Figure 7:**
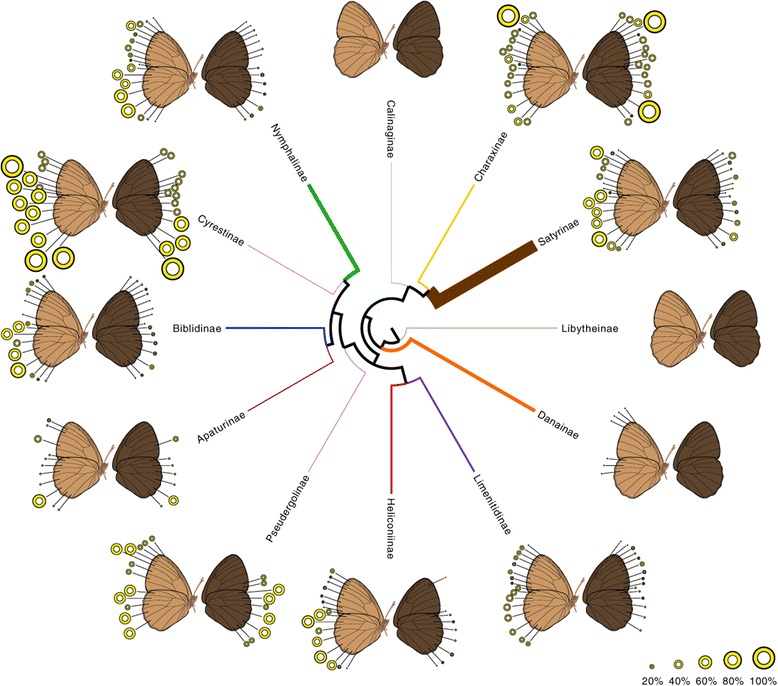
**Frequency of eyespot presence in each wing sector for each Nymphalid subfamily.** The size of each eyespot corresponds to the percentage of taxa sampled with an eyespot present in females in the given wing sector. Eyespots that are never present in any of the taxa sampled are not shown; no eyespots are present in the families Calinaginae and Libytheinae. The width of each branch is proportional to the number of taxa sampled per subfamily. Ventral surface is light brown; dorsal surface is dark brown.

Eyespot deployment to novel locations appears to occur by the same processes of gene network redeployment to novel wing sectors, independently within each subfamily. Despite the variation in eyespot distribution among different Nymphalid subfamilies (Figure 7), none of the results suggest any differences in patterns of ventral eyespot redeployment among nymphalid subfamilies, tribes, and subtribes. The few cases of non-independence in dorsal eyespot origins are also consistent among all lineages examined in that they show that, for both wings, positional information for eyespots Rs, M1, M2, M3, and Cu1 on the dorsal surface evolved from that coding for homologs on the ventral surface. Our results, so far, show no differences between nymphalid lineages. It therefore appears that the evolution of novel positional information for the eyespot gene regulatory network evolved in a parallel fashion across lineages.

### Developmental hypotheses for eyespot redeployments

The developmental mechanisms that underlie eyespot redeployment are not currently understood. Molecular work in model species has illustrated that alterations to enhancers of key genes are often responsible for the deployment of serial homologs in different places in the body, and to evolution of serial homolog number [20]. Those key genes control the activation of the whole gene network that regulates the development of each serial homolog [20]. Two types of modification to the enhancers have been described in various *Drosophila* lineages. The first includes elimination of distinct enhancers of a key regulator of trichome development, *shavenbaby*, leading to loss of trichomes in specific areas of the larval body [6,7]. The second involves modifications to pre-existent single enhancers of *scute* that extended this gene’s expression domain to more anterior body locations, and increased the number of bristles in those areas [5,7]. These alternative modes for variation in serial homolog number can lead to different patterns of correlations regarding gains and losses of serial homologs. The first mode predicts that gains and losses of serial homologs should not be evolutionary correlated with each other, as the enhancers were completely independent of each other, whereas gains and losses of serial homologs in the second mode predicts some form of correlation across serial homologs, i.e., gains (or losses) of new homologs only happen contingent on the presence of a pre-existent serial homolog. It is possible that similar modification to enhancers of eyespot network genes led to the different patterns of redeployment observed in our study.

When eyespots first originated it is possible that the activating signals for network deployment may have been present across every single wing sector. These signals could have involved morphogens diffusing from the veins and from the wing margin as previously proposed [11]. However, it also possible that negative regulators of the network, present in particular wings, surfaces, and/or sectors may have initially prevented the network from being expressed in those areas. This would be the case if binding sites for these negative regulators were initially present in eyespot network genes, at the time of eyespot origins. Over time, however, the removal of these negative regulatory interactions may have allowed the network to become active in additional wings (forewings), surfaces (dorsal surface), and sectors.

Adjacent sectors on an insect wing look developmentally homogeneous, but in fact, they are not. During wing development in *Drosophila*, each wing sector expresses different combinations of transcription factors that gives that sector a unique genetic identity [21,22]. The same applies to dorsal and ventral surfaces and fore and hindwings that express different selector genes [23-25]. For example, the transcription factor Spalt, expressed in the center of the *Drosophila* wing, is also expressed in the butterfly wing, in a sector-specific pattern [26]. Regulatory evolution of the genes in the eyespot network (losing or gaining binding sites for genes such as Spalt, as well as for other wing sector-restricted genes, activators or repressors) may have produced the pattern of successive eyespot origins uncovered in this study. Future studies should examine the regulatory composition of transcription factors expressed in each sector of a nymphalid wing. This information could help us make sense of the particular history of eyespot origins as well as help in the design of experiments to understand the molecular basis of eyespot network deployment.

### Comparison with evolution of serial homology in other systems

Butterfly eyespots are a relatively recent example of serial homology in arthropods. Other serial homologous traits with earlier origins, such as arthropod limbs and body segments, vary far less than eyespots: segment number is stable in most high-rank clades of arthropods, including insects [27], and arthropod limb number has largely remained stable during the past 250 million years [28]. Butterfly eyespot number, on the other hand, continues to evolve and to vary among closely related taxa, and this recent variation may yield clues to its genetic basis.

The ancestral condition for eyespot distribution on the wings also contrasts sharply with other, closely studied serially homologous traits such as arthropod limbs and vertebrate teeth, which are more likely to have arisen as multiple repeated units that later become reduced in number and individuated [2,3]. The evolution of eyespot number appears to be most similar to the evolution of the paired vertebrate limbs, where fins (limbs) appear first in the anterior part of the body (pectoral fins) and later appear in a more posterior region (pelvic fins) [1].

Other serially homologous traits that vary among closely related extant insects include the bristles and trichomes in *Drosophila.* In these species, the genetic basis of variation across closely related species is better understood. Sensory bristles originated in four rows on the thorax in the ancestor of *Drosophila* and other higher Diptera [29]. The lineage leading to *Drosophila* lost some anterior bristles but a few members of the clade subsequently re-evolved them [5,30]. This secondary gain of bristles was due to cis-regulatory evolution at the *scute* locus [5,31]. The recent and independent loss of trichomes, hair-like projections that appear on the larval body in multiple lineages of *Drosophila,* was also due to cis-regulatory evolution at a single locus, *shavenbaby* [7,32]. The evolution of trichomes and their genetic underpinnings has been studied in a phylogenetic context, and ancestral patterns of trichome distribution have been proposed [33], but ancestral trichome distributions on different body locations have not yet been reconstructed.

Nymphalid eyespots represent the reverse of the scenario found for *Drosophila* bristles, in that bristles originally appeared in four complete rows, whereas eyespots arose in a few wing sectors on the ventral hindwing and later originated in new locations, on the forewing and on the dorsal wing surface. Continuing advances in the systematics of higher Diptera [34], stronger hypotheses for ancestral trichome distributions, and identification of the genetic underpinnings of butterfly eyespot development [14] will facilitate the integration of developmental and phylogenetic data to produce a complete understanding of the evolution of serial homology through both loss and gain (redeployment) of individuated traits.

## Conclusions

In butterflies of the family Nymphalidae, eyespots first arose in only a few wing sectors on the ventral wing surface. Over tens of millions of years, the eyespot gene network redeployed to new wing locations; this happened independently in multiple lineages. However, our results show that eyespot gene network redeployment followed the same pattern in all subfamilies. The first eyespots, located in five wing sectors on the ventral hindwing, redeployed to the ventral forewing. From there, these ventral eyespots redeployed to novel locations within the same wing surface, and to the dorsal surface.

### Availability of supporting data

The data sets supporting the results of this article are included within the article (and its additional files).

## Additional files

Additional file 1:
**The presence/absence matrices used for all analyses.**
Additional file 2: Table S1. The clades (tribes or subtribes) containing at least two genera with 13 or more eyespots visible in females. These clades were used for analysis in this study. Additional file 3:
**The log likelihood values and p-values recovered from all model comparisons.** Legend: hind-fore: hindwing to forewing; fedo: female dorsal; feve: female ventral; mado: male dorsal; mave: male ventral; ven-dor: ventral to dorsal wing surface; fefo: female forewing; fehi: female hindwing; mafo: male forewing; mahi: male hindwing; fedofo: female dorsal forewing; fedohi: female dorsal hindwing; fevefo: female ventral forewing; fevehi: female ventral hindwing; madofo: male dorsal forewing; madohi: male dorsal hindwing; mavefo: male ventral forewing; mavehi: male ventral hindwing. Additional file 4: Table S2. Significant correlations between eyespots on the hindwing and eyespots on the forewing. Additional file 5: Table S3. Significant correlations between eyespots on the ventral surface and eyespots on the dorsal surface. Additional file 6: Table S4. Significant correlations between eyespots on the same wing surface.

## References

[1] Ruvinsky I, Gibson-Brown JJ (2000). Genetic and developmental bases of serial homology in vertebrate limb evolution. Development.

[2] Stock DW, Jackman WR, Trapani J (2006). Developmental genetic mechanisms of evolutionary tooth loss in cypriniform fishes. Development.

[3] Gebelein B, Culi J, Ryoo HD, Zhang W, Mann RS (2002). Specificity of Distalless repression and limb primordia development by abdominal Hox proteins. Dev Cell.

[4] Usui K, Goldstone C, Gibert J-M, Simpson P (2008). Redundant mechanisms mediate bristle patterning on the Drosophila thorax. Proc Natl Acad Sci U S A.

[5] Marcellini S, Simpson P (2006). Two or four bristles: functional evolution of an enhancer of scute in Drosophilidae. PLoS Biol.

[6] McGregor AP, Orgogozo V, Delon I, Zanet J, Srinivasan DG, Payre F (2007). Morphological evolution through multiple cis-regulatory mutations at a single gene. Nature.

[7] Sucena É, Delon I, Jones I, Payre F, Stern DL (2003). Regulatory evolution of shavenbaby/ovo underlies multiple cases of morphological parallelism. Nature.

[8] Monteiro A (2008). Alternative models for the evolution of eyespots and of serial homology on lepidopteran wings. BioEssays.

[9] Stathopoulos A, Levine M (2005). Genomic regulatory networks and animal development. Dev Cell.

[10] Gilbert SF, Bolker JA (2001). Homologies of process and modular elements of embryonic construction. J Exp Zool.

[11] Nijhout HF (2001). Elements of butterfly wing patterns. J Exp Zool.

[12] Held LI (2012). Rethinking butterfly eyespots. Evol Biol.

[13] Oliver JC, Beaulieu JM, Gall LF, Piel WH, Monteiro A (2014). Nymphalid eyespot serial homologues originate as a few individualized modules. Proc Biol Sci.

[14] Oliver JC, Tong X, Gall LF, Piel WH, Monteiro A (2012). A single origin for nymphalid butterfly eyespots followed by widespread loss of associated gene expression. PLoS Genet.

[15] Force A, Cresko WA, Pickett FB, Proulx SR, Amemiya C, Lynch M (2005). The origin of Subfunctions and modular gene regulation. Genetics.

[16] Oliver J, Beaulieu J, Gall L, Piel W, Monteiro A: Data from: Nymphalid eyespot serial homologs originate as a few individualized modules. Dryad Digit Repos 2014. http://dx.doi.org/10.5061/dryad.f55c5 10.1098/rspb.2013.3262 PMC4071533 24870037

[17] Wahlberg N, Leneveu J, Kodandaramaiah U, Peña C, Nylin S, Freitas AVL (2009). Nymphalid butterflies diversify following near demise at the Cretaceous/Tertiary boundary. Proc R Soc B.

[18] Maddison WP, Maddison DR: Mesquite: a modular system for evolutionary analysis, version 2.75. [http://mesquiteproject.org] 2013.

[19] Pagel M, Meade A: BayesTraits, version 1.0. [http://www.evolution.rdg.ac.uk/BayesTraits.html] 2013.

[20] Arnosti DN (2003). Analysis and function of transcriptional regulatory elements: insights from Drosophila. Annu Rev Entomol.

[21] Blair SS (2007). Wing vein patterning in Drosophila and the analysis of intercellular signaling. Annu Rev Cell Dev Biol.

[22] Monteiro A, Prijs J, Bax M, Hakkaart T, Brakefield PM (2003). Mutants highlight the modular control of butterfly eyespot patterns. Evol Dev.

[23] Carroll SB, Gates J, Keys DN, Paddock SW, Panganiban GE, Selegue JE (1994). Pattern formation and eyespot determination in butterfly wings. Science.

[24] Weatherbee SD, Nijhout HF, Grunert LW, Halder G, Galant R, Selegue JE (1999). Ultrabithorax function in butterfly wings and the evolution of insect wing patterns. Curr Biol.

[25] Tong X, Hrycaj S, Podlaha O, Popadic A, Monteiro A (2014). Over-expression of Ultrabithorax alters embryonic body plan and wing patterns in the butterfly Bicyclus anynana. Dev Biol.

[26] Monteiro A (2015). The origin, development, and evolution of butterfly eyespots. Annu Rev Entomol.

[27] Minelli A, Maruzzo D, Fusco G (2010). Multi-scale relationships between numbers and size in the evolution of arthropod body features. Arthropod Struct Dev.

[28] McShea DW (1996). Perspective: metazoan complexity and evolution: is there a trend?. Evol (N Y).

[29] Culi J, Modolell J (1998). Proneural gene self-stimulation in neural precursors: an essential mechanism for sense organ development that is regulated by notch signaling. Genes Dev.

[30] Pistillo D, Skaer N, Simpson P (2002). scute expression in Calliphora vicina reveals an ancestral pattern of longitudinal stripes on the thorax of higher Diptera. Development.

[31] Stern DL, Orgogozo V (2008). The loci of evolution: how predictable is genetic evolution?. Evol (N Y).

[32] Stern DL, Frankel N (2013). The structure and evolution of cis-regulatory regions: the shavenbaby story. Philos Trans R Soc Lond B Biol Sci.

[33] Arif S, Murat S, Almudi I, Nunes MDS, Bortolamiol-Becet D, McGregor NS (2013). Evolution of mir-92a underlies natural morphological variation in Drosophila melanogaster. Curr Biol.

[34] Lambkin CL, Sinclair BJ, Pape T, Courtney GW, Skevington JH, Meier R (2013). The phylogenetic relationships among infraorders and superfamilies of Diptera based on morphological evidence. Syst Entomol.

